# Type I restriction enzymes and their relatives

**DOI:** 10.1093/nar/gkt847

**Published:** 2013-09-24

**Authors:** Wil A. M. Loenen, David T. F. Dryden, Elisabeth A. Raleigh, Geoffrey G. Wilson

**Affiliations:** ^1^Leiden University Medical Center, P.O. Box 9600, 2300 RC, Leiden, The Netherlands, ^2^EastChem School of Chemistry, University of Edinburgh, West Mains Road, Edinburgh EH9, 3JJ, Scotland, UK and ^3^New England Biolabs Inc., 240 County Road Ipswich, MA 01938-2723, USA

## Abstract

Type I restriction enzymes (REases) are large pentameric proteins with separate restriction (R), methylation (M) and DNA sequence-recognition (S) subunits. They were the first REases to be discovered and purified, but unlike the enormously useful Type II REases, they have yet to find a place in the enzymatic toolbox of molecular biologists. Type I enzymes have been difficult to characterize, but this is changing as genome analysis reveals their genes, and methylome analysis reveals their recognition sequences. Several Type I REases have been studied in detail and what has been learned about them invites greater attention. In this article, we discuss aspects of the biochemistry, biology and regulation of Type I REases, and of the mechanisms that bacteriophages and plasmids have evolved to evade them. Type I REases have a remarkable ability to change sequence specificity by domain shuffling and rearrangements. We summarize the classic experiments and observations that led to this discovery, and we discuss how this ability depends on the modular organizations of the enzymes and of their S subunits. Finally, we describe examples of Type II restriction–modification systems that have features in common with Type I enzymes, with emphasis on the varied Type IIG enzymes.

## TYPE I RESTRICTION ENZYMES

### Introduction

In the early 1960s, Werner Arber and Daisy Dussoix (1,2) provided evidence that degradation and methylation of DNA lay behind a phenomenon called ‘host-controlled variation in bacterial viruses’, reported a decade earlier (3–5) and reviewed by Luria (6). ‘Variation’ referred to the observation that one cycle of growth of bacterial viruses (also called (bacterio)phages) on certain hosts affected the ability of the progeny phage to grow on other bacterial hosts, by either restricting or enlarging their host range. Unlike mutation, this change was readily reversed, and one cycle of growth in the previous host returned the virus to its original form. ‘Host-controlled variation’ came to be known by the more familiar terms, ‘restriction’ (R) and ‘modification’ (M). It was learned that modification of the cell’s DNA by methylation protected the DNA, whereas the absence of modification on the phage DNA rendered it sensitive to restriction by endonucleases. Restriction–modification (R–M) systems of all types were investigated in the same way, initially, by measuring the efficiency of plating (eop) of phage on alternate bacterial hosts (2,7–10); see references (6,11–15) for early reviews. The genes responsible for restriction and modification were found on bacterial chromosomes, on ‘resistance transfer factor’ plasmids and on the chromosomes of certain temperate phage themselves (11,12,14–16). Their products, restriction endonucleases (REases) and modification methyltransferases (MTases) began to be purified in the late 1960s and have been studied intensively ever since.

Differences in subunit composition, co-factor requirements and DNA-cleavage properties led to the early division of REases (14,17–19) into Type I (*Escherichia coli* EcoKI, EcoBI) and Type II (EcoRI, HindII). Subsequently, Type III enzymes (EcoP1I, EcoP15I) and the Type IV modification-dependent REases (Mcr and Mrr) were also recognized to be distinct classes (18–20). See Restriction Enzyme dataBASE (REBASE) (http://rebase.neb.com) (21) and accompanying papers in this journal. Sequencing and biochemistry have since led to several subdivisions within the Type I and the Type II classes, and boundaries are beginning to blur (20,22).

In the late 1970s, a further difference between Type I and Type II REases was demonstrated by the Linn and Yuan laboratories. Using purified EcoBI and EcoKI, respectively, they showed that Type I enzymes translocated DNA powered by ATP hydrolysis. They reported the formation of DNA loops visible by electron microscopy (EM) and considered reaction intermediates. Concomitant with a large conformational change, these enzymes appeared to translocate the DNA while remaining attached to their recognition sites (23,24).

Viruses and other mobile genetic elements (MGE) have evolved multiple strategies to evade REases, including acquiring modification from their host; synthesizing their own MTases, or hyper-modifying their DNA by incorporating unusual bases; synthesizing antirestriction proteins that mimic DNA; and avoiding specific recognition target sequences (25–27). These processes enable phages to become resistant to the REases they encounter, and once this occurs, those REases no longer protect the host from that particular phage. The solution to this decline in protection over time is for cells to change REase specificity periodically. To do this successfully, however, the specificity of the MTase must change simultaneously in exactly the same way or the new REase will restrict the improperly modified cellular DNA, and the cell will die. Unlike conventional Type II R–M systems that generally cannot do this, Type I R–M systems have evolved an efficient way to change both REase and MTase specificities harmoniously, by using a common DNA sequence-recognition (S) subunit for both restriction and modification.

### Genes, families and distribution

Type I R–M systems are encoded by three genes, termed *hsd* for *h*ost *s*pecificity *d*eterminant: *hsdR* encodes the restriction (R) subunit, *hsdM* the modification (M) subunit and *hsdS* the recognition (S for specificity) subunit. The Type I enzymes studied in most detail are EcoKI, from the workhorse of molecular biology, *E. coli* K12, and the plasmid-encoded EcoR124. The EcoKI genes are located near those for McrBC and Mrr in a cluster called the ‘immigration control region’, a highly divergent locus with alternative DNA segments containing Type I R–M systems, and linked to *serB* in most *E. coli* isolates between the *yjiS* and *yjiA* genes (28–32). Related Type I enzymes in enteric bacteria often localize to this same region, with few exceptions (e.g. EcoR124).

The current division of Type I enzymes into five families, A-E, is based on complementation tests, antibody cross-reactivity and amino acid (aa) sequence; see accompanying paper and reviews (33–37). The enzymes from *E. coli* K12 and *E. coli* B are the founder members of Type IA, EcoAI of *E. coli* 15T^−^ is the prototype of Type IB, plasmid-encoded EcoR124 is the prototype of Type IC, and *Salmonella* StySBLI and *Klebsiella* KpnBI represent Type ID and IE, respectively. Further families undoubtedly exist.

Genome sequencing over the last 15 years, coupled with bioinformatics analysis, reveals that Type I R–M systems are present in approximately one-half of all bacteria and archaea. REBASE maintains a comprehensive list of R–M components of all kinds, both characterized (i.e. biochemically confirmed), putative (unconfirmed) and decayed (disrupted aa sequence). At recent count, of 2145 sequenced bacterial and archaeal genomes in REBASE, 1140 (53%) have at least one each of the *hsdR*, *M* and *S* genes needed for a Type I system. In all, 835 genomes (39%) appear to have no *hsd* genes at all, and the remaining 170 genomes (8%) have some *hsd* genes, but perhaps not all. Disruptions and scrambled gene organizations are common.

Among the 1140 genomes that include Type I R–M systems are ∼2100 putative *hsdR* genes, 2200 putative *hsdM* genes and 2600 putative *hsdS* genes—or roughly two of each per organism. The multiplicity of systems varies considerably though. Most genomes have only one Type I system, but two, three or more is common, with as many as eight in some bacteria (e.g. *Desulfococcus oleovorans*). Some species of *Mycoplasma* have only one or two *hsdR* and *hsdM* genes but multiple *hsdS* genes: e.g. 10 in *Mycoplasma pneumoniae*, 13 in *Mycoplasma suis* and a record 22 in *Mycoplasma haemofelis*. In these organisms, different *hsdS* genes probably cycle on and off to effect a continuously changing defense against invaders (38,39). The individual target recognition domains (TRDs) of S subunits can shuffle into different combinations by genetic rearrangements, and so the number of different R–M specificities that these microbes can conjure up is potentially far larger than the number of *hsdS* genes they possess. Nearly 500 different specificities, and perhaps twice that number, could emerge from 22 *hsdS* genes, a remarkable defensive repertoire.

### Enzyme activities

Type I R–M enzymes are pentameric proteins of composition 2R+2M+S. They require ATP, Mg^2+^ and S-adenosylmethionine (SAM) for activity and display both REase and MTase activities. A trimer of 2M+S acts solely as an MTase (35,40,41). Characteristically, Type I enzymes recognize bipartite DNA sequences comprising two half-sequences separated by a gap—for example, AACNNNNNNGTGC (AAC N6 GTGC) where *N* = any base (Table 1). This stems from the repetitive organization of the S subunit that comprises two separate TRDs, one for recognizing each half-sequence. Recombination between TRDs generates new sequence specificities and is a powerful driver of Type I R–M system diversification.

**Table 1.** Characterized wild-type Type I R–M enzymes, arranged chronologically

Enzyme	Family	Recognition sequence	Me-interval	Reference
**EcoBI**	IA	TG**A** N8 **T**GCT	8	(42–44)
**EcoKI**	IA	A**A**C N6 G**T**GC	8	(45)
**EcoAI**	IB	G**A**G N7 G**T**CA	9	(46,47)
**StyLTIII**	IA	G**A**G N6 R**T**AYG	8	(48)
**StySPI**	IA	A**A**C N6 G**T**RC	8	(48)
**EcoDI**	IA	TT**A** N7 G**T**CY	8	(49)
**EcoDXXI**	IC	TC**A** N7 R**T**TC	8	(50)
**EcoR124I**	IC	GA**A** N6 R**T**CG	7	(51)
**EcoEI**	IB	G**A**G N7 A**T**GC	9	(52)
**CfrAI**	IB	GC**A** N8 G**T**GG	9	(53)
**EcoprrI**	IC	CC**A** N7 R**T**GC	8	(54)
**StySKI**	IB	CG**A**T N7 G**T**TA	9	(55)
**StySBLI**	ID	CG**A** N6 **T**ACC	6	(56)
**NgoAV****^a^**	IC	GC**A** N8 **T**GC	8	(57)
Eco377I		GG**A** N8 A**T**GC	9	(58)
Eco585I^b^		GCC N6 TGCG	?	(58)
Eco646I		CC**A** N7 CTTC	?	(58)
Eco777I		GG**A** N6 TATC	?	(58)
**KpnBI**	IE	CAA**A** N6 R**T**CA	7	(59)
**KpnAI**	ID	GA**A** N6 **T**GCC	6	(60)
StySEAI		AC**A** N6 **T**YCA	6	(60)
StySGI		TA**A**C N7 R**T**CG	9	(60)
Eco394I		G**A**C N5 R**T**AAY	7	(61)
Eco826I		GC**A** N6 C**T**GA	7	(61)
Eco851I		GTC**A** N6 **T**GAY	6	(61)
Eco912I		C**A**C N5 **T**GGC	6	(61)
**CsaII**		CC**A**C N6 C**T**C	8	(62)
**VbrI**		AGH**A** N7 **T**GAC	7	(62)
**VbrII**		CT**A**G N6 R**T**AA	8	(62)
**CjeFII**		CA**A**Y N6AC**T**	9	(62)
**CjeFIV**		TA**A**Y N5 **T**GC	6	(62)
**BceSVI**		TA**A**G N7 **T**GG	8	(62)
**EcoGIV**		CC**A**C N8 **T**GAY	9	(63)
**MpuII**		G**A** N7 **T**AY	7	(64)
**SauMW2I**		CC**A**Y N5 TTAA	?	(65)
**SauMW2II**		CC**A**Y N6 TGT	?	(66)
**SauN315I**		**A**TCN5CC**T**	9	(66)
**SauN315II**		CC**A**Y N6 G**T**A	8	(66)

^a^The S subunit of NgoAV is truncated, resulting in symmetric specificity.

^b^No adenine occurs in the first haf-sequence of Eco585I implying that cytosine becomes methylated instead.

Column 1: The name of the prototype enzyme; isoschizomers are not listed. Bold type signifies that the system is well-characterized. Column 2: Complementation group to which the enzyme belongs, if known. Column 3: Only one strand of the recognition sequence is shown, oriented 5′–3′. For the well-characterized enzymes, the sequence is shown in the orientation for which the first (5′) half is specified by the N-TRD of the S subunit, and the second (3′) half is specified by the C-TRD. For the remaining systems, the TRD assignment is not known, and the orientation shown is arbitrary. The numeral in the recognition sequence indicates the number of non-specific bases between the two half-sequences; ‘AAC N6 GTGC’, for example, means AACNNNNNNGTGC. Bases in bold type indicate the positions of methylation if known, generally one A in the 5′ half-sequence and another that is the complement of the T in the 3′ half-site. Column 4: Number of base pairs between the two methylated bases. Enzymes belonging to the same family have similar spacing. Column 5: Publication reporting the specificity.

#### REase and translocase activities

The R subunit is essential for REase activity. It contains an N-terminal endonuclease domain fused to a so-called ‘motor’ domain found in many other DNA and RNA processing enzymes with translocation or helicase activity (66–74). The R subunit contacts the M_2_S MTase core via the M subunits. If neither half of the recognition sequence is methylated, the R subunits translocate the flanking DNA and cleave it a variable distance away (75), approximately midway between neighboring recognition sites (76), while the enzyme remains attached with the site. This behavior generates DNA loops visible by EM (23,24) and atomic force microscopy (77,78). Mutations in the endonuclease domain can prevent DNA cleavage without affecting DNA translocation function (66).

Single-molecule studies coupled with improved biochemical and biophysical methods have illuminated the DNA translocation properties of EcoKI and EcoR124 and revealed hitherto unsuspected details about protein dimerization and DNA looping (78). Dimerization appears to be favored when the DNA molecule contains two recognition sites, while DNA looping can occur in the absence of ATP hydrolysis. This way of bringing distant DNA regions together using complex protein assemblies may be a common phenomenon. Magnetic tweezers experiments have provided details of the biophysical aspects of translocation, including the rate of translocation of DNA by the molecular motors—the R subunits—as well as their processivity and ATP dependence (79–83). These studies showed that the two motors could work independently, and that the enzyme tracked along the helical pitch of the DNA on torsionally constrained molecules. Apparently, translocation could stop and restart by disassembly and reassembly. This finding might be of interest to those working on the much larger complexes that contain the related eukaryotic DNA helicases and chromatin-remodeling proteins of the SNF2 superfamily (67). Further studies confirmed the model that ATP hydrolysis is coupled to bidirectional DNA translocation, which would explain the DNA loops seen in the EM by Bob Yuan and colleagues 25 years earlier (24,84,85). The R subunits disengage transiently roughly every other 500 bp using ATP, but translocation progresses ∼2 kb. Once translocation is blocked, either by collision with another molecule or the presence of supercoiled DNA, the DNA is cleaved. Also, there is *in vitro* evidence for cleavage at a replication fork (86). Though the enzyme remains attached to the DNA, it can be displaced by other translocating enzymes such as the major recombination complex of *E. coli*, RecBCD (84).

#### MTase activity

Methylation of the recognition sequence is catalyzed by both the M_2_S trimer (M.EcoKI) and the R_2_M_2_S pentamer (EcoKI), and it requires the co-factor SAM. Usually, methylation converts one adenine in each half-sequence to *N*6-methyladenine (m6A) (Table 1). Like other MTases (87–90), M.EcoKI flips the target base out of the DNA helix to carry out methyl transfer (91). M.EcoKI and related Type IA enzymes are maintenance MTases, with a preference for hemimethylated substrates in which only one of the two half-sequences is methylated (92). However, both the small Ral protein of phage *λ* and mutations in the N-terminus of the M subunit can turn M.EcoKI into a *de novo* MTase (93,94), the same activity as found for the Type IB enzyme EcoAI (46).

### Enzyme structure

**Figure 1.**
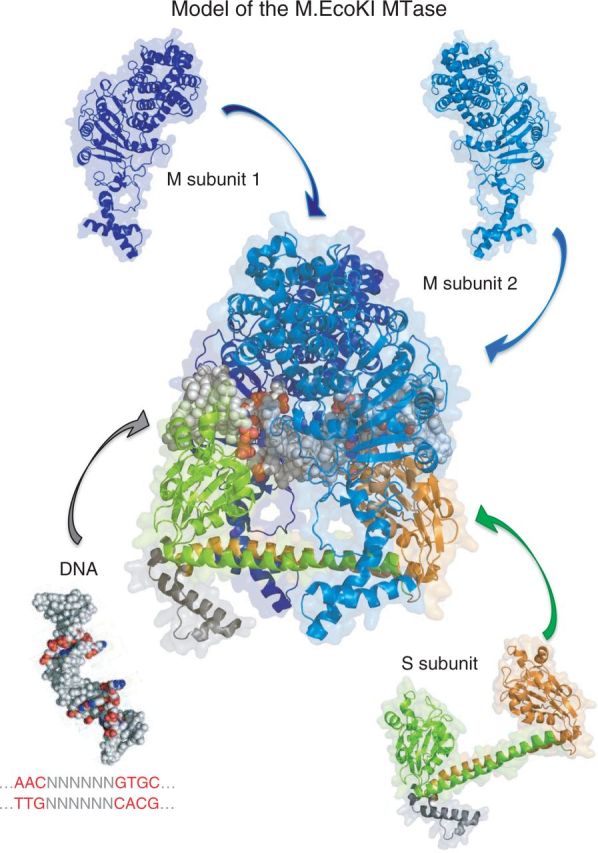
Model of the M.EcoKI MTase (pdb file 2Y7H). The S subunit is composed of two TRDs in inverted orientations. Each TRD comprises a globular DNA-binding domain and an alpha helical dimerization domain. The N-TRD (green) in this protein is specific for the sequence AAC (the 5***′***-half-sequence), and the C-terminal domain (orange) is specific for GCAC (the 3***′*** half-sequence). Zipper-like association of the helices separates the globular domains by a fixed distance and reverses the orientation of the C-TRD, resulting in the composite recognition sequence that is bipartite: AAC N6 GTGC. Each TRD also associates with one M subunit (identical, but shown here in different shades of blue for clarity) to form an M_2_S trimer. Neither S nor M subunits bind to DNA alone, but the trimer binds specifically at the recognition sequence and catalyzes methylation of one adenine in each half-sequence. Because the TRDs are inverted, the two M subunits have opposite orientations. Consequently, both strands of the recognition sequence become methylated, the ‘top’ strand of the 5***′*** half-sequence (Am6AC) and the ‘bottom’ strand of the 3***′*** half-sequence (GCm6AC). This enables the DNA of the host cell to be distinguished from infecting DNA during DNA replication.

Atomic structures of Type I R–M enzymes have been difficult to obtain. Crystal structures of individual subunits have been solved, but not of complexes. S subunit structures were solved in 2005 (pdb:1YF2 and 1YDX) (95,96) and 2011 (pdb: 3OKG) (97), and M subunit structures in 2005 and 2007 (pdb:2AR0 and 2OKC; New York Center for Structural Genomics, and Joint Center for Structural Genomics, unpublished). These structures did not include DNA, but models of the M.EcoKI trimer with DNA (Figure 1) and with a DNA-mimic protein, Ocr, were reported in 2009 (40) based on EM single particle reconstructions (pdb:2Y7H and 2Y7C).

**Figure 2.**
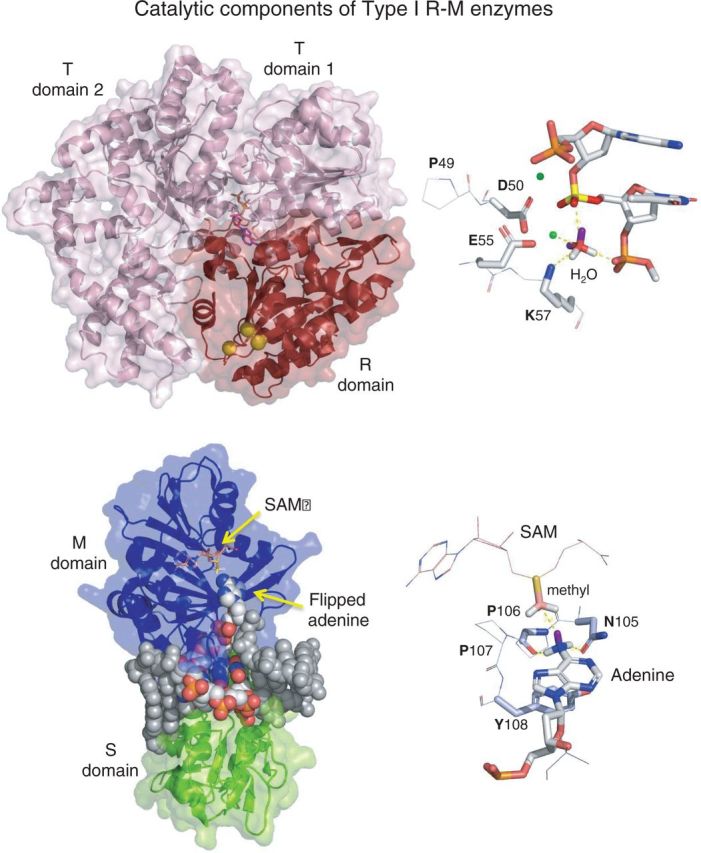
Catalytic components of Type I R–M enzymes. Upper figure, left: The R/T subunit of EcoR124I (pdb: 2W00). The R domain (red), responsible for DNA cleavage, comprises the N-terminal ∼260 aa. It contains the common PD-D/EXK endonuclease catalytic site, composed of D151, E165 and K167 (yellow spheres). Upper figure, right. The PD-D/EXK catalytic site of the Type II REase, MvaI (pdb:2OAA). D50 and E55 coordinate divalent metal ions (in this case two Ca^2+^ ions, shown as green spheres at reduced scale for clarity). The hydrolytic water molecule is oriented by interaction with a metal ion, the general base K57, and a phosphate oxygen from the adjacent base. These interactions position a lone pair electron orbital (purple sticks) of the water molecule for in-line nucleophilic attack on the phosphorus atom (bright yellow), initiating the DNA cleavage reaction. Catalysis occurs in the presence of Mg^2+^, but not in the presence of Ca^2+^; hence, this structure represents the pre-cleavage complex. Lower figure, left: The monomeric γ-MTase, M.TaqI, with bound DNA (pdb: 1G38). SAM was absent in this complex, which represents the pre-methylation complex. SAM has been added here through modeling by structural alignment with pdb:2ADM. Lower figure, right: The catalytic NPPY of M.TaqI is composed of N105, P106, P107 and Y108. When the target adenine is flipped into the catalytic site, the hydrogens of the 6-amino group form hydrogen bonds with the side chain amide carbonyl of N104 and the main chain carbonyl of P105. These lie below the plane of the base and likely induce the nitrogen to switch from the planar sp^2^ orbital configuration it normally possesses, to the tetrahedral sp^3^ configuration (105). In this latter configuration, the lone pair orbital of nitrogen (purple stick) is appropriately positioned for in-line nucleophilic attack on the carbon thiol (pink) of SAM, initiating the DNA methylation reaction (105,106).

The first crystal structure of a Type I R subunit (EcoR124) was published in 2009 [pdb: 2W00; (98,99)]. The catalytic site for DNA-cleavage in these proteins belongs to the ‘PD-(D/E)XK’ endonuclease superfamily, the same kind found in many Type II restriction endonucleases [(100–104); Figure 2]. The motor domain belongs to the RecA-like ATP-dependent family, a clade of the Additional Strand Conserved E family (107), of which the well-known AAA+ motor proteins are another clade (108). The location of the nuclease domain opposite the translocase domain suggests a coupling between DNA translocation and cleavage. The crystal structure of an N-terminal fragment of a putative Type I R subunit from *Vibrio* sp. has also been reported [pdb:3H1T; (109,110)]. It contains three globular domains with a nucleolytic core and the ATPase site close to the putative site for DNA-translocation.

Together, these structures provided a framework for how the complete pentameric enzyme assembles, and how the motor domains might translocate the DNA. Recently, the complete structure of both the EcoKI and EcoR124I enzymes was elucidated by EM single particle reconstruction studies.

## CONTROL OF R–M ACTIVITY

### Restriction alleviation

Type I R–M systems are suppressed following DNA damage by ultraviolet irradiation, 2-aminopurine and nalidixic acid, a phenomenon termed ‘restriction alleviation’ (RA) (111). Following the damage event, a cell population that normally restricts phage to an eop of ∼10^−^^5^, say, instead restricts to only ∼10^−^^1^. This mechanism is presumed to protect the cell while the damage is being repaired or during homologous recombination (112). During this period, unmodified sites can be created in the host chromosome, and RA prevents these from being restricted. Restriction (but not modification) is also suppressed following transfer of R–M genes to a new host; that is, expression of the new genes does not lead to cleavage of the unmodified host chromosome. Investigation of the dynamics of appearance of restriction activity in the transfer recipient revealed a long delay after entry of the EcoKI *hsdR, M* and *S* genes. This delay was genetically dependent on a host function, at first called HsdC (113,114). Further genetic investigation demonstrated that the delay mechanism was mediated by the ClpX chaperone and its proteolytic form, the ClpXP complex (115).

The two sorts of RA were unified in further experiments, which studied stability, by western blot, of components of the Type IA and Type IB R–M systems (115,116). Degradation occurs specifically when the R subunit is assembled into a translocation-competent complex. Mutations in any of the R subunit DEAD box motifs, which impair ATPase activity and translocation both *in vivo* and *in vitro* (117,118), relieve sensitivity of the R subunit to ClpXP (119). Mutations in the endonuclease motif, in contrast, do not relieve sensitivity to degradation (119).

The EcoR124I Type IC R–M system is insensitive to ClpXP-mediated control but also displays RA (120,121). RA could result from disassembly of the pentameric restriction complex (during endogenous RA when the complex will be attempting to translocate on the host chromosome) or inefficent assembly (during establishment of RM in a new host) for this plasmid-borne system. Still unresolved is the mechanism by which these enzymes distinguish between an invading unmodified DNA and an unmodified new or old host. A recent article showed that the R subunit of EcoKI (but not those of representative Type IB and IC enzymes) can be phosphorylated on threonine. This could play a role in localization, disassembly or proteolysis (122).

### Antirestriction and antirestriction-modification

Antirestriction (antiR) and antirestriction–modification (antiR–M) systems have evolved in many MGEs such as phage, plasmids and transposons. Their activities increase the probability that the MGE will survive the cell’s R–M systems and initiate a successful infection. Phage T4 has the most numerous antiR and antiR–M mechanisms (26,27,123–125). Early work on antiR–M was carried out by the groups of Studier at NIH, Kruger in Berlin, Bickle in Basel (126–131) and by the groups of Belogurov and Zavil’gel’skii in Russia; reviewed in (22,26,27,132–134). The Wilkins laboratory in the UK solved the riddle of control of antiR encoded by the self-transmissible IncI plasmid: the *ardA* gene was transiently induced during conjugation by transcription from a single-stranded promoter (135–138). The evolutionary struggle between antiR–M and R–M has been likened to an ‘arms race’ with new mechanisms and counter-mechanisms developing continuously (22,26,27,132–134,138–140). We describe examples of these later in the text, including alteration of the methylation preference of M.EcoKI and the synthesis of antiR–M proteins: Ocr from phage T7 (141), ArdA from transposon Tn916 of *Enterococcus faecalis* (142), ArdB (143) from a pathogenicity island of *E. coli* CFT073, and KlcA [an ArdB homolog (144)] from plasmid pBP136 of *Bordetella pertussis* (145).

#### De novo methylation

M.EcoKI and related Type IA modification enzymes are ‘maintenance’ MTases that exhibit a strong preference for hemi-methylated recognition sites. This enables the MTase to be highly active on replicating DNA, but very slow to modify foreign unmodified DNA when it enters. This property can be foiled by antirestriction activities. Interaction with the phage lambda Ral protein converts M.EcoKI to a ‘*de novo*’ methyltransferase (93,146). Induction of *ral* (*r*estriction *al*leviation) under the control of an inducible promoter enhanced survival of unmodified phage lambda 800-fold within 1 min. This fast reaction suggested direct interaction of the Ral protein with EcoKI perhaps inducing a conformational change to enhance methylation over restriction (93). Mutations in the *hsdM* gene can achieve the same result, thus effectively converting EcoKI from a maintenance to a *de novo* MTase in the absence of Ral (94). A model (147) was proposed to explain the differences in activities between wild-type and mutant enzymes based on biochemical and structural data (148).

#### AntiR and antiR–M proteins

The Ocr, ArdA and ArdB proteins vary in their occurrence in nature (149–151). Ocr seems to be confined to phage, particularly T7 and its relatives (150–153). In T7, it is encoded by gene *0.3*, the first gene to enter the *E. coli* host on infection. It is synthesized in large amounts for the first 2 min of infection, during which insertion of further T7 DNA halts (154–156). Sufficient Ocr protein is produced in this brief period to completely inhibit the host’s Type I enzymes, following which Ocr synthesis stops and the rest of the phage genome enters the cell in safety. (*In vitro*, Ocr also binds to *E. coli* RNA polymerase (157); whether this is relevant *in vivo* has not been investigated.)

ArdA and ArdB proteins are generally encoded by conjugative plasmids and transposons, and they are also often the first genes to enter the recipient cell (149). As the entering DNA is single stranded and resistant to restriction, an unusual promoter is formed: the DNA of plasmid ColIb-P9 forms a double-stranded hairpin to allow transcription of *ardA* (135–137). Production of ArdA or ArdB leads to rapid inhibition of the host’s Type I enzymes. This novel transcription method may well be more common and deserves further research. Importantly, both *ardA* and *ardB* genes are widespread (as determined by genomic sequencing projects) and frequently accompany antibiotic-resistance genes. Their presence might have a considerable impact on the rate of spread of resistance in bacterial populations.

#### The structure of Ocr

**Figure 3.**
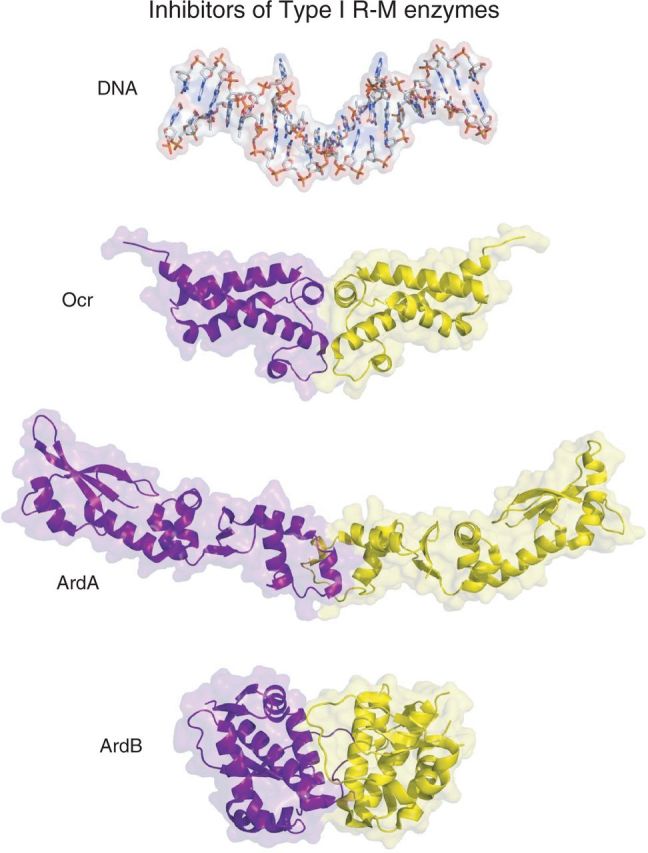
Protein inhibitors of Type I R–M enzymes. Top panel: DNA model (hydrogen atoms omitted) from pdb:2Y7H displayed on the same scale as the proteins for structural comparisons. Panel b: Ocr (pdb:1S7Z and 2Y7C) from bacteriophage T7; panel c: ArdA (pdb:2W82) from Tn916 of *E. faecalis* (117); panel d: ArdB (pdb:2WJ9) from a pathogenicity island of *E. coli* CFT073. All three proteins are homodimeric. Their subunits are identical, but are displayed here in different colors.

Ocr is a striking example of DNA mimicry by a protein. It is a homodimer of two 116-aa subunits (127) that interface by a complementary fit of hydrophobic surfaces [(141,158); pdb:1S7Z and 2Y7C; Figure 3]. The dimer is banana shaped with a length of ∼7.5 nm and a general width of 2 nm thickening to 2.5 nm at the dimer interface (141,159,160). Each monomer contains several well-packed alpha helices, a long loop and flexible N- and C-termini. The narrowness of the structure means that it has a minimal hydrophobic core, which contains a considerable number of aromatic aa, perhaps akin to the aromatic core of another DNA mimic, Qnr (161). Despite this small core, Ocr is stable to heat and chemical denaturation (160). Arrayed on the surface of each monomer are 34 negatively charged aa and only 6 positive aa (∼12 of each would be expected for a typical globular protein of this size), although not all of these are required for activity (162–164). The negative surface charges have roughly the same spacing as the phosphate groups on 24 bp of B-form DNA with a bend in the center, which explains its affinity for Type I enzymes (165).

#### The structure of ArdA

ArdA also mimics B-form DNA in geometry and electrostatics (166). The crystal structure shows ArdA to be a homodimer, but it can exist as both monomer and dimer in solution [(142); pdb:2W82; Figure 3]. The ArdA encoded by Tn916 has 166 aa per subunit; the dimerization interface is smaller than in Ocr. The dimer is a highly elongated bent cylinder ∼15 nm long and 2 nm in diameter along its entire length. The narrowness of the protein again means that it has a minimal hydrophobic core, but unlike Ocr, ArdA is not resistant to denaturation (142). The fold of ArdA is completely different to that of Ocr with each ArdA monomer comprising three small loosely packed domains. The domain folds have been found in other protein structures and comprise a mix of alpha helices, beta strands and loops. The surface of each monomer is covered with numerous carboxyl groups such that the dimer mimics ∼42 bp of bent B-form DNA. The domain organization and low stability of ArdA imply structural flexibility and suggest that the protein might mold itself to the contorted, S-shaped, DNA-binding groove of Type I enzymes (41), with different domains interacting with the different R, M and S subunits (40).

#### The structure of ArdB

The structures of two members of the ArdB family have been solved by crystallography [ArdB from *E. coli* CFT073 (143); pdb:2WJ9; Figure 3] and nuclear magnetic resonance spectroscopy [KlcA from *B.pertussis* (145); pdb:2KMG]. The proteins are close homologs with >30% aa sequence identity. *Klc* genes form part of the *kor* operon involved in the regulatory network of these promiscuous plasmids (144). The ArdB structures are different from those of Ocr and ArdA, being small, rather normal-looking, globular proteins although with a novel polypeptide fold. They are neither elongated nor possess significant charged patches so are unlikely to cause antiR via DNA mimicry. KlcA is a monomer in solution, but the ArdB structure is a dimer with two intermolecular disulphide bridges and a well-packed hydrophobic interface. As the cysteine residues are not conserved, dimer-formation may not be required for activity and may not even arise in the reducing environment of the bacterial cytoplasm. The novel fold of ArdB shows two alpha helices projecting out from the protein with a smaller third helix nearby completing a domain. This domain is then ‘cupped’ or held in the palm of a hand by another domain comprised of helices, beta strands and loops. The absence of conserved charged patches or a narrow structure to allow association with a DNA-binding site suggests a different mechanism of inhibition from ArdA and Ocr, although it is possible that the ArdB structures could be different when in the cytoplasm.

#### Effect on restriction and modification

The effectiveness of Ocr, ArdA and ArdB in inhibiting restriction and modification by Type I enzymes has usually been investigated *in vivo* by comparing the eop of phage on restriction-proficient hosts with or without antiR–M genes (139,141,142,145,158,167). Active antiR enhances the number of recovered phage, and these in turn can then be tested for modification by their eop on restriction-proficient and -deficient strains. Quantitative comparisons in these experiments are difficult due to varying experimental details, but the *in vivo* titration assay, in which an inducible promoter and antiR–M expression is controlled by inducer concentration, recently introduced by the group of Zavil’gel’skii, suggests a good way forward for quantitative comparison of antiR–M systems (139,158,167).

The eop assays show that Ocr effectively blocks both restriction and modification by all Type I families. This is a direct consequence of the extremely strong binding of Ocr to the DNA-binding groove in the MTase core (170). ArdA and ArdB block restriction in all Type I families but are much less effective at blocking modification (139,141,145,158,167). This minimal anti-M activity is due to the binding of ArdA to the MTase core being of similar or weaker strength than DNA binding to the core. Although the binding is weak, it is sufficient to prevent restriction. ArdB also shows little or no anti-M effect *in vivo*, and no interaction has been observed *in vitro* between ArdB and the MTase core. Furthermore, although ArdB causes antiR *in vivo*, no effect could be demonstrated *in vitro* on restriction. Therefore, the mechanism of antiR used by ArdB is indirect rather than the binding mechanisms used by Ocr and ArdA. It would be useful to define an active core for ArdB activity by constructing truncated variants of the protein.

Our understanding of antiR–M is still in its infancy. Aside from the three systems described earlier in the text, few others have been studied beyond their initial discovery. Given their synergistic role with R–M systems in regulating horizontal gene transfer and the ‘resistome’ (171,172), this deficiency in our knowledge needs to be addressed.

#### EcoprrI, an apoptosis enzyme

An especially elaborate example of defense and counter-defense concerns EcoprrI*.* The *prrABD* genes encode a conventional Type IC R–M system (54). The *prrC* gene, embedded in this operon, encodes a latent anticodon nuclease (ACN) that sacrifices the host on phage infection. PrrC is normally sequestered within the Type I enzyme complex, but it is released to act when phage T4, which is completely resistant to Type I REases, infects the host. Lethal damage is inflicted on the infected cell and expression of phage proteins is inhibited, by cutting the anticodon loop of tRNA^lys^. T4 counters the DNA/RNA restriction activity of *EcoprrABCD* with three dedicated T4 proteins (173), T4 polynucleotide kinase and RNA ligase that repair the cleaved tRNA, and Stp (174) that inhibits the Type I activity but liberates the ACN in the process [see (175) for review].

Interestingly, ACN appear to be involved in stress responses in both prokaryotic and eukaryotic cells: e.g. DNA repair defects sensitize yeast to ACN activity (176), and angiogenin induces tRNA cleavage in mouse cells (177). Such an ‘RNA-based innate immune system that distinguishes self from non-self’ (178) may also be considered programmed cell death after infection (179).

## TYPE I DNA SEQUENCE SPECIFICITY

### Characterization

Few Type I R–M enzymes were known to exist before the advent of genome sequencing, and these were confined to *E. coli* and close bacterial relatives. Even today, the number of characterized Type I enzymes is small compared with Type II enzymes (Table 1). This disparity reflects the difficulty of identifying Type I enzymes and determining their recognition sequences. R–M systems of all types were studied in the same way at first, by their effects on plating efficiencies of phages. As the enzymology of restriction and modification began to be explored in the 1970s, DNA-cleavage assays of bacterial cell extracts largely replaced phage-plating assays. This led to the discovery of many new Type II REases, as these cleave DNA at fixed positions and produce discreet DNA fragment patterns, but it had little effect on the discovery of Type I enzymes (19). Type I REases cleave DNA at variable positions with respect to their recognition sites, and so they do not produce signature fragment patterns. Consequently, they cannot be identified easily in cell extracts, and their recognition sites are far more difficult to map.

By 1980, only two Type I enzymes had been discovered—EcoBI (42–44) and EcoKI (45)—compared with 56 different Type II REases (180). Eight more Type I specificities were discovered in the 1980s [versus over 100 new Type II REases (181)] and another two in the 1990s (Table 1)—together with some remarkable variant specificities that had arisen by chance or by design (Table 2). Fourteen more Type I specificities were discovered early in the following decade, due mainly to work in Junichi Ryu’s laboratory based on the restriction of sets of sequenced plasmids during transformation (58–61), and 11 more have been added very recently from work elsewhere (62,63,65,192). The original systems are well-characterized for the most part, but little is known yet about the newer ones. Characterizing Type I enzymes would likely have remained difficult but for a development that is poised to revolutionize this field. Next-generation sequencing of genomic DNA provides a rapid way of determining sequence specificity based on the sites of methylation rather than the sites of cleavage (61,62).

**Table 2.** Derivative Type I R-M systems with altered specificities

Enzyme	Recognition sequence	S subunit organization	Reference
StySB	G**A**G N6 R**T**AYG	[GAG]∼[CRTAY]	
StySP	A**A**C N6 G**T**RC	[AAC]∼[GYAC]	
**StySQ**	A**A**C N6 R**T**AYG	[AAC]∼[CRTAY]	(48,180–182)
**StySJ**	G**A**G N6 G**T**RC	[GAG]∼[GYAC]	(185)
EcoAI	G**A**G N7 G**T**CA	[GAG]∼[TGAC]	
StySKI	CG**A**T N7 G**T**TA	[CGAT]∼[TAAC]	
**SKI/AI**	CG**A**T N7 G**T**CA	[CGAT]∼[TGAC]	(55)
EcoR124I	GA**A** N6 R**T**CG	[GAA]∼[CGAY]	
EcoDXXI	TC**A** N7 R**T**TC	[TCA]∼∼[GAAY]	
**EcoDR2**	TC**A** N6 R**T**CG	[TCA]∼[CGAY]	(186)
**EcoRD2**	GA**A** N6 R**T**TC	[GAA]∼[GAAY]	(186)
**EcoDR3**	TC**A** N7 R**T**CG	[TCA]∼∼[CGAY]	(186)
**EcoRD3**	GA**A** N7 R**T**TC	[GAA]∼∼[GAAY]	(186)
EcoR124I	GA**A** N6 R**T**CG	[GAA]∼[CGAY]	
**EcoR124II**	GA**A** N7 R**T**CG	[GAA]∼∼[CGAY]	(51,187)
EcoR124I	GA**A** N6 R**T**CG	[GAA]∼[CGAY]	
**EcoR124IΔ50**	GA**A** N7 **T**TC	2[GAA]	(188)
EcoDXXI	TC**A** N7 R**T**TC	[TCA]∼∼[GAAY]	
**EcoDXXIΔC**	TC**A** N8 **T**GA	2[TCA]	(189)
**EcoDXXIΔN**	GA**A**YN5 R**T**TC	2[GAAY]	(190)
EcoAI	G**A**G N7 G**T**CA	[GAG]∼[TGAC]	
**EcoAI cp380**	G**A**G N7 G**T**CA	[TGAC]∼[GAG]	(191)

Column 1: Enzymes in regular type are parental; these are also listed in Table 1. Enzymes in bold are derivatives. Column 2: The recognition sequence is printed in the orientation for which the 5′ half-sequence is specified by the N-TRD, and the 3′ half-sequence is specified by the C-TRD. Numerals indicate the number of non-specific bases between the two half-sites. Bold type indicates the bases known or inferred to be methylated. Column 3: Inferred compositions of the S subunits. The specificities of the individual TRDs are given in the order in which they occur in the S subunit, e.g. ‘[GAG]∼[CRTAY]’, indicates that the N-TRD recognizes GAG, and the C-TRD recognizes CRTAY. ‘∼’ indicates that the TRDs are joined into a single protein chain. ‘∼∼’ indicates that this linkage contains additional aa that increase their separation. S subunits that are homodimers of a single TRD are depicted as ‘2[GAA]’, for example, meaning that it comprises two molecules of a GAA-specific TRD. Because the orientation of the second TRD in S subunits is inverted with respect to the first TRD, the 3′ half-sequence is always the ‘complement’ of the specificity of that TRD. Column 4: Publication reporting the derivative.

Single-Molecule Real-Time (SMRT) DNA-sequencing instruments of the kind manufactured by Pacific Biosciences not only distinguish the bases from one another but also distinguish their modification states, differentiating m6A from adenine, and m4C, hm5C and to some extent m5C, from cytosine (64). As modification always takes place within the recognition sequence in all types of R–M systems, identifying the sequence contexts in genomic DNA that contain modified bases reveals the methylation profile of the organism—its ‘methylome’—and the specificities of all of its active Mtases (193). Bioinformatics analysis of the genome reveals the MTases that are present and their likely characteristics, and then it becomes a matter of correctly matching the MTases to the DNA sequences methylated.

Methylation data have begun to emerge from SMRT sequencing projects and are growing rapidly (62,63,168,169,192). New R–M specificities of various types have already been uncovered, and REBASE has added a new section to track the accumulating information (http://rebase.neb.com/rebase/rebase.charts.html>Specialized Information> PacBio). Because multiple R–M systems as well as solitary MTases are often present in each genome, sub-cloning and methylome re-analysis will be needed in many instances to confirm which specificity belongs to which system. In addition, for Type I enzymes, there is the added task of establishing which TRD of the two present in each S subunit recognizes which half of its recognition sequence. Amino acid sequence comparisons combined with experiments of the kinds described later in the text suggest ways in which these latter assignments can be made. Structural similarities between crystallized Type I S and M subunits (40,95,96), *γ*-class adenine MTases (105), and Type IIG RM enzymes (194,195), coupled with our growing understanding of how these proteins recognize DNA specifically (196), make it plausible that by the end of this decade, we will be able to ‘decode’ the specificities of many Type I enzymes by simply inspecting the aa sequences of their S subunits.

### Type I S subunits

**Figure 4.**
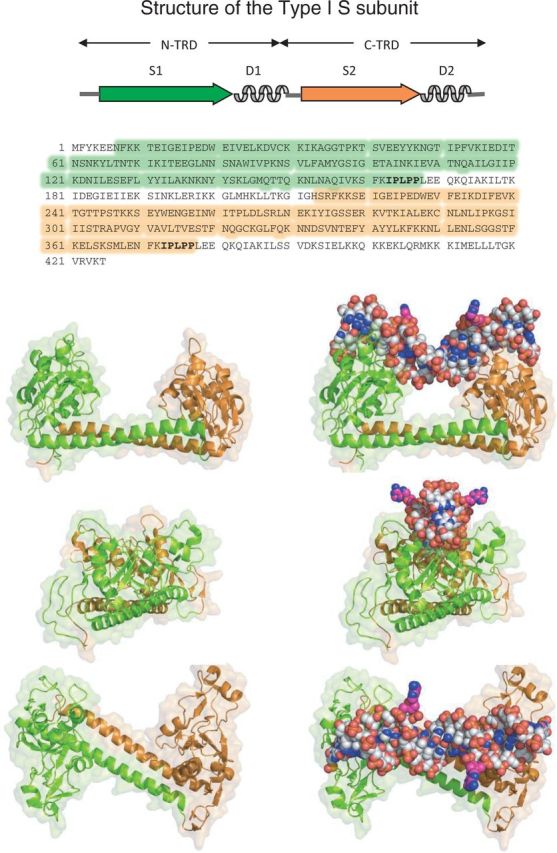
Structure of the Type I S subunit (pdb:1YF2). The recognition sequence of this protein, S-MjaXI, from *Methanocaldococcus* (formerly Methanococcus) *jannaschii* is not known. It is closely related to the EcoKI-family (Type IA) of enzymes depicted in Figure 1. The upper diagram shows the domain organization of the protein; arrows represent DNA-binding domains, and curly lines represent dimerization alpha helices. The aa sequence of the protein is shown below, with the domains in corresponding colors. Below this are three views of the structure, from three perpendicular directions, ‘sideways’, ‘end-on’, and ‘above’. The panels on the left depict the protein; those on the right depict the protein with modeled DNA positioned approximately as it is bound. The DNA was taken from pdb:2Y7H and transferred by structural alignment of the S subunits.

**Figure 5.**
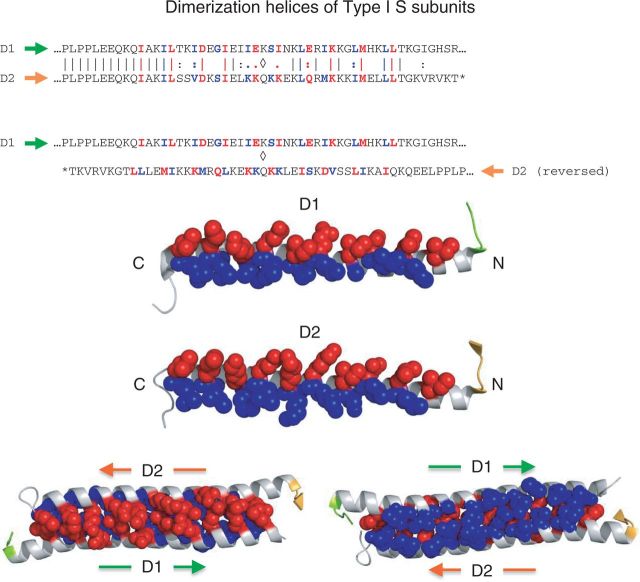
Dimerization helices of Type I S subunits. Upper diagram: aa sequence alignment of the dimerization helices of S-MjaXI (pdb:1YF2; D1 and D2 in Figure 3) show that they are similar but not identical (top). The proline-rich motif that precedes each helix (IPLPP) is a hallmark of Type I S subunits and likely plays a structural role establishing the correct architectural relationship between the S and D domains. The helices interact in opposite orientations to form an antiparallel coiled-coil (bottom). Adjacent pairs of aa (red and blue) form the dimerization interface and occur with the 4-3-4-3 … spacing characteristic of leucine zippers. Middle diagram: Exposed dimerization surfaces of two helices. Side chains of red aa point ‘up’ toward the bound DNA, and those of blue aa point ‘down’. To form the coiled coil, D1 must be rotated 180 degrees around the vertical axis and docked against the surface of D2 shown. Lower diagram: Within the coiled coil, red aa from one helix interdigitate with red aa from the other helix, forming the ‘upper’ surface of the coiled coil, the surface closest to the bound DNA (left). And blue aa from one helix inter-digitate with blue aa from the other helix to form the ‘lower’ surface on the other side (red). Within the coiled-coiled, red side chains from one helix stack on blue side chains from the other helix in an alternating pattern. At this interface, the helices have complimentary topologies such that a ridge or bump in one is accommodated by a valley or depression in the other, resulting in a close hydrophobic fit.

The S subunits of Type I enzymes are responsible for DNA sequence recognition. They have a duplicated organization comprising two TRDs in tandem (Figure 4). Each TRD specifies one half of the bi-partite recognition sequence. The TRDs consist of a globular specificity domain (S) and an alpha-helical, ‘dimerization’ domain (D). The TRDs occur as direct repeats in the linear aa sequence, but they assume inverted orientations in the folded protein as the helices associate (‘dimerize’) in antiparallel to make a coiled-coil ‘leucine-zipper’ (Figure 5). In simplest form, the N-terminal TRD (N-TRD) of the S subunit has the composition S1-D1, the C-terminal TRD (C-TRD) has the composition S2-D2, and the entire S subunit has the composition S1-D1∼S2-D2. In some Type I families, circular permutation of this organization alters the positions of these domains.

### Specificity changes

#### TRD exchanges

The structure of S subunits enables the specificities of Type I R-M systems to change spontaneously and robustly. The first observation of such a change was reported in 1976. Using phage transduction to transfer chromosomal DNA from a strain of *Salmonella* that expressed the Type I SP system (now StySPI) to a derivative that expressed the SB system (now StyLTIII), a transductant was recovered that expressed a novel specificity, ‘SQ’ (182). With considerable insight, the authors attributed this new system to a genetic cross-over in the middle of the parental *hsdS* genes.

Heteroduplex analysis of the *hsdS* genes showed the same organization as other members of the EcoKI family (197), consisting of an N-terminal variable region (=S1), a central conserved region (=D1), a second variable region (=S2) and another conserved region (=D2) at the C-terminus. The SQ *hsdS* gene was a hybrid consisting of the N-TRD of the SP gene joined to the C-TRD of the SB gene (183,184). Independently, the recognition sequence of StySQ was determined and also found to be a hybrid comprising the 5′ half-site of SP and the 3′ half-site of SB (Table 2) (48,198).

These experiments suggested strongly that specificity was determined by the variable regions of the S subunits, and that each specified one-half of the recognition sequence independently of the other. The finding that discrete protein domains recognized discrete DNA sequences was a revelation at the time and marked a significant advance in our understanding of how proteins interact with DNA. To complete matters, the reciprocal hybrid was intentionally created by genetic recombination and was shown to have the reciprocal specificity. In this system, ‘StySJ’, the N-TRD came from SB, and the C-TRD from SP, and as predicted, its specificity combined the 5′ half-site of SB with the 3′ half-site of SP (Table 2) (185). The nucleotide sequences of the parental and hybrid S genes confirmed their structures and showed that crossing-over had occurred in a 130-nt stretch of near sequence identity that we now know encodes the first of the two dimerization helices (184,185).

StySB and StySP belong to the Type IA family, but domain shuffling is not confined to enzymes of this group. The S subunits of other Type I families also have a tandemly repeated organization of alternating specificity and dimerization domains and are similarly suited to exchange these by recombination. A hybrid S subunit constructed *in vitro* by joining the N-TRD of StySKI to the C-TRD of EcoAI—both members of the Type IB family—displayed a hybrid specificity and enabled the specificities of the individual domains to be assigned (55). Likewise, reciprocal hybrids between the Type IC enzymes EcoR124I and EcoDXXI were constructed and shown to possess the expected hybrid specificities (Table 2) (186). There are no reports of hybrids between members of different Type I families, but there are indications that this occurs in nature, such as the N-TRDs of StyLTIII and EcoEI (55).

#### Gap length changes

Enzymes of the Type IC family also change specificity by unequal crossing-over at a short sequence-repeat in D1, the first of two helices that connect the DNA-binding domains. The length of these helices determines the separation between the two specificity domains (Figure 4), and this in turn determines the gap in the recognition sequence—or more precisely, the separation between the two bases that becomes methylated. Studies in several laboratories showed that EcoR124I and a variant, EcoR124II, recognize the same DNA sequence half-sites, but differ in how far they are apart: EcoR124I is specific for a gap of 6 bp (GAA N6 RTCG), and EcoR124II is specific for a gap of 7 bp (GAA N7 RTCG) (187,199–201). This change was found to depend on a 12 bp sequence in conserved domain D1 that was repeated twice in EcoR124I but three times in EcoR124II (51,187,201). The repeat codes for Thr-Ala-Glu-Leu (TAEL)—just over one alpha helical turn—and the extra repeat in EcoR124II moves the two S domains 0.34 nm further apart and rotates them by 36 degrees, similar to the offset between adjacent bp in duplex DNA (187). Two other enzymes, EcoDXXI and EcoPrrI, also contain three TAEL repeats in D1, and this appears to be the optimum number based on aa sequence alignments (190). During the construction of hybrids between EcoR124I and EcoDXXI, the number of TAEL repeats was also changed, and the hybrid recognition sequences were found to change in precisely the manner predicted (Table 2) (186).

The crystal structures of putative S subunits show that the alpha helices encoded by the dimerization domains interconnect to form an antiparallel coiled coil (95–97). Amino acid side chains down their lengths interlock like tines of a zipper and form a hydrophobic core that holds the two helices together (Figure 5). Addition or loss of a turn in one helix but not in the other, due to a change in the number of TAEL repeats, must change the register between of the aa in the zipper because, if it did not, the gap would not change. How this perturbation is accommodated is not clear. Introducing a compensatory change in the other helix to restore the original register would be worth investigating.

#### Homodimeric S subunits

In the changes described earlier in the text, the order of the specificity domains remained the same, one N-TRD being swapped for another N-TRD, and so on. Experiments in the 1990’s showed that the order makes little biological difference because, as they adopt inverse orientations in the folded protein, the S subunit is rotationally symmetric. This is due to the antiparallel nature of the coiled coil: it both separates the TRDs and inverts their orientations. In hindsight, a structure with rotational symmetry was only to be expected: the S subunit must orient the two M subunits to methylate different strands of the recognition sequence so that both can become methylated. As the strands of DNA have opposite orientations, the M subunits must also have opposite orientations, and because they derive their orientations from their association with the S domains, these must have opposite orientations as well. That this was indeed the case emerged from experiments with EcoR124I.

A mutant of EcoR124I was isolated, HsdS(Δ50), that lacked ∼160 aa from the C-terminus of the S subunit, including most of the C-TRD. This mutant was active, and it was found to have a new specificity, GAA N7 TTC, that was a palindrome of the sequence recognized by the N-TRD (Table 2) (188). The fact that this sequence was bipartite, like regular Type I enzymes, indicated that a second molecule of the N-TRD was substituting for the missing C-TRD, and that the two TRDs were therefore interchangeable. It also indicated that the TRDs have inverted orientations, and that they need not be joined in a continuous polypeptide chain to function together. Perhaps more surprising is that the helices forming the coiled coil can also substitute for each other. D1 forms an antiparallel zipper with D2 in the normal ‘heterodimeric’ S subunit. In HsdS(Δ50), D1 evidently zippers with itself, and does so in a conservative way that preserves the parental 7-nt separation between methylated adenines. Considerable sequence similarity exists between the dimerization domains of Type IC S subunits, perhaps explaining why one helix can satisfactorily replace the other.

A comparable truncation arose by insertion of transposon Tn5 into the C-TRD of the EcoDXXI S subunit, and likewise resulted in a homodimer specific for a palindrome of the N-TRD specificity (Table 2) (189). The separation between the methylated bases—8 nt in this case due to the presence of a third TAEL repeat—was also preserved. To test whether the C-TRD could substitute for the N-TRD in the reverse scenario, the N-terminus of the EcoDXXI S subunit was intentionally deleted. This mutant behaved in the same way as the other homodimers and recognized an interrupted palindrome of the C-TRD specificity (190). When the N- and C-terminal-truncated S genes were co-expressed to create a mixture, wild-type EcoDXXI activity was regenerated, confirming further that the two TRDs need not be joined into a single protein chain to work together. That they are joined in all of the characterized Type I systems and most of the putative ones, too, suggests that fusion nevertheless confers a selective advantage. Fusion fixes the TRD stoichiometry to 1:1 and limits the combinatorial pairing possibilities when multiple TRDs are present in the same cell.

One natural Type I system has been found that has a palindromic specificity: NgoAV (Table 1). The S gene of this system is fragmented into several open reading frames (ORFs). The first ORF encodes a complete N-TRD similar in size and composition to the EcoR124I(Δ50) and EcoDXXI truncations described earlier in the text, and it evidently behaves in the same way. The C-terminal domain is incomplete and inactive (57). EcoR124I, EcoDXXI and NgoAV all belong to the Type IC family. No equivalent homodimers have been reported for enzymes from other Type I families. The D1 and D2 helical domains of Type IA S subunits have little sequence similarity, suggesting they might not be able to substitute for each other as can those of the Type IC S subunits.

#### Circular permutation of S subunits

Within Type I families, the S subunits have a consistent organization, but between families, they are circular permutations of one another. For Type IA S subunits, the domain order is S1-D1-S2-D2, whereas for Type IB S subunits, it is D2-S1-D1-S2. The Type IC S subunits are in-between, beginning and ending within D2. These forms differ in where, within a circle of alternating specificity and dimerization domains, the N- and C-termini occur. That these forms are structurally equivalent was demonstrated by circularly permuting the EcoAI S subunit, a Type IB family member (191). In one permutation, most of the N-terminal conserved domain was moved to the C-terminus, mimicking the organization of Type IC S subunits. This construct, cp91, was active in both methylation and restriction. From the equivalent position in the central conserved region, a second permutation was constructed, cp380, in which the entire N-terminal half of the protein was switched to the C-terminus, reversing the order of the TRDs. This construct, a Type IC mimic in reverse, was active in only methylation but, tellingly, it displayed the same EcoAI specificity as its parent, confirming the inverted orientations of the two domains that make up the S subunit. Two other permutations, one a mimic of Type IA S subunits, the other a Type IB mimic but reversed, proved to be inactive (191). Evidently, the permuted forms are not necessarily functionally equivalent. The S domain order seems not to matter internally, but the positions of the N- and C-termini do matter, perhaps affecting proper folding or interaction with the M and R subunits or resistance to proteases (191).

Indications that S domains switch order in nature can be inferred from aa sequence comparisons. The first specificity domain of StySKI (S1), for example, specifies CGAT. A similar domain occupies the second position (S2) in EcoR124I where it specifies a similar sequence CGAY (55). Other possible examples can be found among the uncharacterized systems in GenBank and REBASE (202).

#### Recognition sequence orientations

Most of the natural and derivative Type I specificities are asymmetric, reflecting the ‘heterodimeric’ composition of the S subunit. Because of this asymmetry, Type I recognition sequences can be written in two ways depending on which strand is referenced. EcoKI, for example, can be written as ‘AAC N6 GTGC’ or as ‘GCAC N6 GTT’. Ideally, these sequences should all be oriented in the same, biologically meaningful, way where the 5′ half-site is specified by the N-TRD of the S subunit, and the 3′-half site is specified by the C-TRD. However, deciding which strand should be used for the sequence in any instance requires first knowing the specificities of those TRDs; without this information, the orientation is arbitrary. Nonetheless, because the TRDs have inverted orientations in the S subunit, the specificity of the C-TRD is not the sequence of the second half-site of the recognition sequence, but rather its complement. This becomes important when comparing the specificities of TRDs that occupy different positions, such as the N-TRD of StySKI and the C-TRD of EcoR124I, referred to earlier in the text (55).

## RELATIVES OF TYPE I SYSTEMS

Type I systems are large complex R–M enzymes, which at first encounter can be puzzling. Why three subunits? Why is the S subunit internally duplicated while the other subunits are not? Why is the stoichiometry 1S:2M:2R? This organization begins to make sense when Type I enzymes are viewed from the perspective of variations on a theme, or more accurately variations on two themes, one being the inviolable necessity to protectively modify both DNA strands of the recognition sequence, and the other being the benefit derived from coupling R and M activities to the same sequence-specificity determinant. Type I enzymes represent only one of many variations on these themes, albeit a prominent one.

### Classes of MTases

Three major classes of amino-MTases occur in prokaryotes, termed alpha, beta and gamma (203,204). Members of all three classes catalyze the transfer of a methyl group from SAM to the exocyclic amino group of adenine, forming m6A. Many members of the α and β classes methylate the exocyclic amino group of cytosine instead of adenine, to form m4C. MTases of the *γ* class methylate mainly adenine, and only rarely [e.g. the unusual M.NgoMXV and its homologs (205,206)] methylate cytosine instead. A further major class of MTases occurs in both prokaryotes and eukaryotes—the m5C-MTases. These exclusively methylate carbon-5 of the cytosine ring to form 5-methylcytosine (m5C), and they are catalytically distinct from the amino-MTases. Bioinformatics analysis suggests that additional kinds of amino-MTases might exist, representing minor classes (204). The m4C-specific MTase M.MwoI, for example, has been proposed to be a member of the delta class based on differences with M.SfiI (207), which is a β-class m4C-MTase, and M.TvoORF1413P has been proposed to be member of the zeta group (208).

### Gamma-class MTases

Gamma-class MTases are the most diverse of the three major amino-MTase classes. They comprise a 250–450-aa catalytic domain and a sequence-specificity domain that is either fused to form the C-terminus of a single protein (M∼S) or is present as a separate subunit (M+S). A curious mechanistic difference distinguishes these groups in that the *γ*-MTases extract the base to be methylated from one DNA strand, whereas the α− and β-MTases extract it from the other strand. This difference can be seen by comparing the DNA co-crystal structure of the *γ*-MTase M.TaqI (pdb:1G38) (105), for example, with that of the α-MTase T4Dam (pdb:1YFL) (209). Type I enzymes belong to the *γ*-class, and a consistent strand-specificity is evident in their recognition sequences. Invariably, the base that becomes methylated in the 5′ half-sequence is located in the ‘top’ strand, and the base that becomes methylated in the 3′ half-sequence is located in the ‘bottom’ strand.

In aa sequence alignments of *γ*-MTases, two motifs stand out: motif I, which forms the SAM binding site, and motif IV, which is typically Asn-Pro-Pro-aromatic [i.e. NPP(Y/F/W)] and forms the catalytic site for methyl transfer. The four catalytic aa are located in a slot in the surface of the protein into which the target base flips before methyl transfer. The aromatic aa stacks with the flipped base, compensating for the loss of DNA base stacking (210), and the exocyclic amino group forms two hydrogen bonds with the protein, compensating for the loss of the Watson–Crick base-pairing hydrogen bonds (105,106) (Figure 2). Methyl transfer occurs directly, without formation of a covalent protein–DNA intermediate such as occurs in the m5C-MTases.

**Figure 6.**
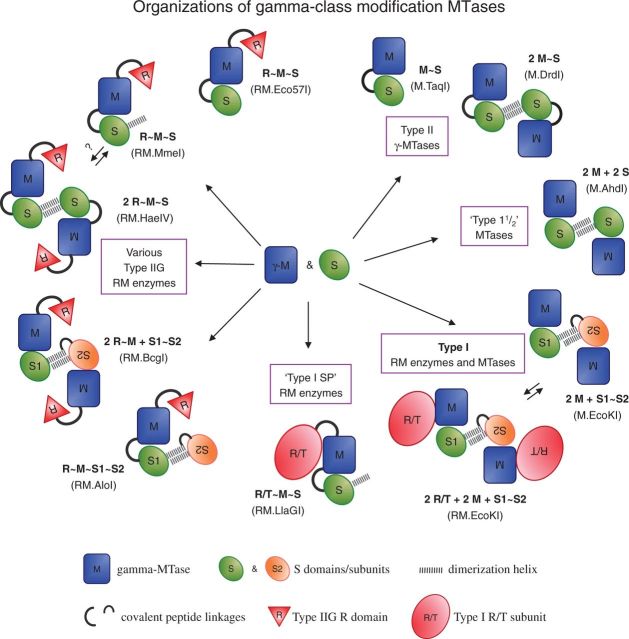
Organizations of gamma-class modification MTases. γ-MTases catalyze transfer of a methyl group from SAM to the exocyclic amino group of adenine (or in rare cases, cytosine) at specific target sequences in duplex DNA. Their organizations vary depending on whether they methylate both DNA strands, or only one, and whether they cleave the DNA instead if the sequence is completely unmodified. At minimum, γ-MTases comprise an M component for base flipping and methyl transfer (blue oblongs), and an S component for sequence-recognition (green and orange ovals). These can be separate subunits or discrete domains. S components can function individually, or as inverted pairs that associate through antiparallel dimerization helices. The members of such pairs can be identical, in which case the structure is homodimeric, or they can differ, in which case the structure is functionally heterodimeric albeit usually connected into a single protein chain. Type I and Type IIG R–M enzymes are γ-MTases that also restrict DNA. For Type I enzymes, a supplementary R/T subunit (large red ovals) catalyzes endonuclease and DNA-translocase activities. For Type IIG enzymes, an R domain (red triangle) is permanently present at the N-terminus. The latter combine with S components in configurations that mirror those of the monofunctional γ-MTases. The figure is not exhaustive; it depicts only the commonest variants of γ-MTases but reveals a close evolutionary connection between Type I R–M systems and certain Type II R–M systems. An example of each organization is given in parentheses.

Gamma-class MTases form the M_2_S core of Type I R–M systems, and also the MTase components of many Type II R–M systems. Consequently, they represent an evolutionary bridge between these two types of R–M systems (Figure 6). We discuss later in the text some of the varied organizations in which the *γ*-MTases occur.

#### Monomeric γ-MTases

M.TaqI is a typical *γ*-MTase. It is a simple monomer of ∼420 aa comprising an N-terminal catalytic domain (245 aa) connected to a C-terminal specificity domain (165 aa) by a 10-aa linker (Figure 2). M.TaqI is specific for the sequence TCGA. As this sequence is symmetric, M.TaqI can bind to it in either orientation and methylate both of the DNA strands, first one and then the other. This enables M.TaqI to serve as the sole modification component of the Type II TaqI R–M system. M.SalI, M.PstI, M.HincII and many other *γ*-MTases function in the same way in their respective Type II systems (Table 3). With the recent, and unpublished, exception of MmeI, M.TaqI is the only *γ*-MTase whose crystal structure has been solved bound to DNA (105,211,212).

**Table 3.** Varied organizations of *γ*-MTases found in Type II R–M systems

Enzyme	Recognition sequence	Enzyme composition
M.TaqI	**T**CG**A**	*γ*M∼[TCG**A**]
M.SalI	G**T**CG**A**C	*γ*M∼[GTCG**A**C]
M.HincII	G**T**YR**A**C	*γ*M∼[GTYR**A**C]
M.PstI	C**T**GC**A**G	*γ*M∼[CTGC**A**G]
M.Eco57I	C**T**GA**A**G	*γ*M∼[CTNN**A**G]
M1.Eco31I	GG**T**CTC	*γ*M∼[GAG**A**CC]
M.Esp3I	CG**T**CTC	*γ*M∼[GAG**A**CG]∼m5C-MTase
M.BseRI	GAGG**A**G	m4C-MTase∼*γ*M∼[GAGG**A**G]
M.XmnI	G**A**A N4 T**T**C	2*γ*M∼[G**A**A]
M.DrdI	G**A**C N6 G**T**C	2*γ*M∼[G**A**C]
M.XcmI	CC**A** N9 **T**GG	2*γ*M∼[CC**A**]
M.BsaBI	G**A**T N4 A**T**C	2*γ*M + 2[G**A**T]
M.PshAI	G**A**C N4 G**T**C	2*γ*M + 2[G**A**C]
M.AhdI	G**A**C N5 G**T**C	2*γ*M + 2[G**A**C]
M.BstXI	CC**A** N6 **T**GG	2*γ*M + [CC**A**]∼[CC**A**]

Differences occur in the number of catalytic domains/subunits (*γ*M), the number of specificity domains/subunits (S) and in their linkage: whether they are separate proteins (+) or joined into a continuous protein chain (∼) Column 1: MTases are differentiated from other components of R–M systems by the prefix ‘M.’. Column 2: Numerals within the sequence indicate the number of non-specific bases between the two half-sequences. Bold type indicates the bases known or inferred to be methylated. ‘**A**’ signifies that the adenine shown is methylated, and ‘**T**’ signifies that the complementary adenine on the other strand is methylated. Most of these enzymes methylate both DNA strands. Column 3: Domain/subunit composition of the MTase. ‘+’ signifies the components are separate subunits, ‘∼’ signifies they are covalently joined. The specificity of the TRD is given in square brackets. ‘[G**A**G]’ means that the TRD recognizes GAG and methylates the A. Some enzymes comprise two MTases fused together; in these cases, the presence of the other MTase is indicated by ‘m4C-MTase’ or ‘m5C-MTase’.

#### DoubleMTases

Many MTases are specific for sequences that are not symmetric. These enzymes can bind to their recognition sequence in one orientation only, and consequently they can methylate only one DNA strand. Such MTases can nevertheless participate in R–M systems by partnering with a complementary MTase that methylates the other strand. Such MTase partnerships comprise the modification components of most Type IIS R–M systems. For example, in the Eco31I/BsaI R–M systems, the modification component consists of a *γ*-MTase partnered with a separate m5C-MTase. In the related Esp3I/BsmBI R-M systems, the modification component is a fusion of a *γ*-MTase and an m5C-MTase (213,214). In the BssSI and BseRI systems, the modification components are fusions of *γ*-MTases and m4C-MTases (Table 3).

#### Homodimeric γ-MTases

An alternative way in which *γ*-MTases with asymmetric specificities participate in R–M systems is through dimerization (215). Instead of partnering with a complementary MTase, these pair with a second molecule in opposite orientation, achieving similar results. These homodimeric MTases act only in this paired state, and their recognition sequences are interrupted palindromes—inverted repeats of the asymmetric specificity separated by a gap. The modification enzymes of most of the Type II R–M systems with bipartite recognition sequences are *γ*-MTases that likely act in this way, including M.XmnI, M.DrdI and M.XcmI (Table 3; Figure 6). No proteins of this kind have been crystallized, and so their structures remain unproven, but their C-terminal specificity domains resemble TRDs of Type I S subunits, including motifs suggestive of coiled-coil dimerization helices.

#### ‘Type 1½’ MTases

The relationship between Type II R–M systems and Type I MTases is even closer for enzymes such as M.BsaBI, M.PshAI and M.AhdI (216). These consist of separate M and S subunits rather than composite M∼S chains, and they are close natural equivalents of the EcoR124I**Δ** and EcoDXXI**Δ** mutants described earlier (Tables 2 and 3; Figure 6). M.AhdI has the composition 2M+2S (216). Small-angle neutron scattering shows that its structure is similar to that modeled for Type I MTases (217). Dubbed ‘Type 1½’ MTases, these enzymes are intermediate between typical Type I and typical Type II *γ*-MTases, and they likely represent the ancestral form from which contemporary Type I enzymes evolved by fusion of previously separate TRDs. Finally, the modification component of the BstXI system is not just closely related to Type I MTases, it appears to be one. M.BstXI comprises separate M and S subunits, but unlike the S subunits of Type 1½ MTases, which are single TRDs, the BstXI S subunit has a duplicated organization with two TRDs. These TRDs are similar in aa sequence but not identical, and evidently, they recognize the same half-sequence, CCA, resulting in a recognition sequence that is symmetric overall (Table 3).

### Type IIG R–M enzymes

Type IIG enzymes [also termed Type IIC; (20)] are *γ*-MTases with N-terminal DNA-cleavage domains (Table 4; Figure 6). They are bi-functional R and M enzymes that cleave at fixed distances from their recognition sequences, one or two turns of the helix away. When first discovered, they were so distinct from other Type II REases that they were assigned to a new class, ‘Type IV’ (218). Subsequent agreement to classify all REases that cut at fixed positions as ‘Type II’, regardless of phylogeny, led to their being re-designated ‘Type IIG’, and ‘Type IV’ was assigned to the modification-dependent enzymes instead (20). Type IIG enzymes are widespread in bacteria and archaea. They are less common than Type I enzymes but more diverse and occurring in several distinct organizations (Table 4). Type IIG enzymes are present in 833 of 2145 sequenced genomes in REBASE, or roughly 40%. In all, 1779 systems are distributed among these genomes with an average multiplicity of two per genome. Most genomes have only one or two Type IIG systems but some have many, and strains of *Borrelia burgdorferi*, the agent of tick-borne Lyme disease, have up to an astonishing 20!

**Table 4.** Varied organizations of Type IIG R-M enzymes

Enzyme	Recognition sequence and cleavage positions	System organization
RM.BpuSI	GGG**A**C 10/14	R∼*γ*M∼[GGG**A**C] & M1 & M2
RM.BseRI	GAGGAG 10/8	R∼*γ*M∼[GAGGAG] & M1∼M2
RM.Eco57I	CTGA**A**G 16/14	R∼*γ*M∼[CTGA**A**G] & M
RM.BpmI	CTGG**A**G 16/14	R∼*γ*M∼[CTGG**A**G] & M
RM.Tth111II	CAARCA 11/9	1-2R∼*γ*M∼[CAARCA]
RM.MmeI	TCCR**A**C 20/18	1-2R∼*γ*M∼[TCCR**A**C]
RM.NmeAIII	GCCG**A**G 20/18	1-2R∼*γ*M∼[GCCG**A**G]
RM.AquIV	GRGGA**A**G 20/18	1-2R∼*γ*M∼[GRGGA**A**G]
RM.BaeI	10/15 **A**C N4 G**T**AYC 12/7	**2R∼***γ***M + S1∼S2**
RM.BcgI	10/12 CG**A** N6 **T**GC 12/10	2R∼*γ*M + S1∼S2
RM.CspCI	10/12 CC**A**C N5 **T**TG 12/10	2R∼*γ*M + [CC**A**C]∼[CA**A**]
RM.BsaXI	9/12 **A**C N5 C**T**CC 10/7	2R∼*γ*M + S1∼S2
RM.NgoAVIII	10/12 TC**A** N5 G**T**C 13/11	2R∼*γ*M + [TC**A**]∼[G**A**C]
RM.HaeIV	7/13 G**A**Y N5 R**T**C 14/9	2R∼*γ*M∼[G**A**Y]
RM.AloI	7/12 GG**A** N6 G**T**TC 12/7	R∼*γ*M∼[GG**A**]∼[GA**A**C]
RM.PpiI	8/13 G**A**G N5 G**T**TC 12/7	R∼*γ*M∼[G**A**G]∼[GA**A**C]
RM.TstI	7/12 GG**A** N6 G**T**G 13/8	R∼*γ*M∼[GG**A**]∼[C**A**C]

Differences occur in the number of catalytic domains/subunits (RM), the number of specificity domains/subunits (S) and in the linkages between them. Frequently, these mirror the different forms of *γ*-MTases listed in Table 3. Column 1: Type IIG enzymes are *γ*-MTases with N-terminal DNA-cleavage domains. They are differentiated from other system components by the prefix ‘RM.’. For brevity, this prefix is often omitted. Column 2: Numerals inside the sequences indicate the number of non-specific bases between the half-sequences. Numerals outside indicate the positions of cleavage. GGGAC 10/14, for example, signifies that cleavage takes place to the right of the sequence shown, 10 bases further down on that strand and 14 bases further down on the other strand. Column 3: Organization of the system, and domain/subunit composition of the R-M enzyme. ‘∼’ indicates the components are covalently joined; ‘+’ indicates they are separate subunits. ‘&’ indicates that additional MTases (M, M1, M2) are components of the system and provide protective modification. Stoichiometry, known or inferred, is indicated by the numeral preceding the protein. MmeI-like enzymes are monomers but likely act as homodimers/homotetramers; this bimodal behavior is indicated by ‘1–2’. Where known, the specificities of the TRDs are given in square brackets and are shown in the order in which they occur within the protein. When the order is not known, the TRDs are designated S1 and S2.

The DNA-cleavage domains of Type IIG enzymes comprise approximately the N-terminal 200 aa and belong primarily to the ‘PD-(D/E)XK’ family of endonucleases, the same as found in Type I R subunits, and the commonest among the Type IIP REases. The Asp and Glu residues of this motif, in combination with a phosphate oxygen and water molecules, coordinate one or two divalent metal ions, typically magnesium or manganese. These ions are essential for catalysis and are thought to counter the build up of negative charge on the phosphorus during the transition state. They might also induce a positive charge on the phosphorus atom before catalysis. The Lys acts as a general base by deprotonating a structured water molecule, producing the hydroxide ion that attacks the phosphorus (Figure 2). A single catalytic site is present in Type IIG R domains, and cleavage of duplex DNA likely involves transient dimerization between neighboring enzyme molecules. The crystal structure of only one Type IIG enzyme has been published: BpuSI (GGGAC 10/14; see footnote to Table 4 for convention) (194). The cleavage domain of BpuSI resembles the well-characterized C-terminal cleavage domain of the Type IIS enzyme, R.FokI, and it cleaves DNA with the same staggered geometry, producing 4-base 5′-overhangs (219,220). BpuSI is unusual in this regard, as most Type IIG enzymes create 2-base 3′-overhangs, indicating that their catalytic sites cleave across the minor groove of DNA rather than across the major groove. BpuSI was crystallized without DNA, and comparison with M.TaqI indicates that it must undergo significant structural rearrangements to bind to its recognition sequence and effect catalysis (194).

#### Monomeric Type IIG enzymes: Eco57I

Eco57I (CTGAAG 16/14) was the first member of this new class to be characterized (218). Its large size (997 aa; 117 kDa) is typical for these enzymes and reflects their composite nature of three joined domains, R, M and S (Figure 6). The recognition sequence of Eco57I is asymmetric but continuous (Table 4). The enzyme methylates only one strand and cleaves on only one side, indicating that it binds to the sequence as a monomer in one orientation only. Requisite methylation of the other strand is accomplished by a separate MTase, M.Eco57I, also a *γ*-MTase (218). This latter enzyme has a degenerate specificity (CTNNAG) enabling it to methylate both strands of the sequence, the same A in the top strand methylated by Eco57I, and the sole A in the bottom strand (Table 4). The need for an accompanying MTase confines the evolutionary diversification that Eco57I can undergo to just the central 2 bp. Related enzymes with specificity differences at these positions are known, but they are few in number.

The crystallized BpuSI also acts as a monomer and methylates the single A in the top strand. Two additional MTases accompany this enzyme: a separate *γ*-MTase that methylates the same adenine, and an m5C-MTase that methylates a cytosine in the bottom strand. In the BseRI Type IIG system, the accompanying modification enzyme is a fusion of an m4C-MTase joined to a *γ*-MTase (Table 4) (221). In all of these monomeric Type IIG systems, the accompanying MTases methylate both DNA strands, hinting that the intrinsic methylation activity of the R–M enzymes themselves might play a role other than protective modification.

#### Multimeric Type IIG enzymes: BcgI

BcgI (10/12 CGA N6 TGC 12/10) was discovered shortly after Eco57I and has the novel property of cleaving on both sides of its recognition sequence, releasing a small fragment containing the recognition sequence at every site it cuts (222). BcgI comprises two proteins, an RM subunit and an S subunit (Figure 6), and it represents a trimmed-down fixed-cleaving version of Type I enzymes (223–225). More enzymes of this kind, also termed Type IIB (226), have been discovered and characterized including BaeI, CspCI, BsaXI, SdeOSI and NgoAVIII (227), but they are far less common than Type I enzymes (Table 4). The gap between the two half-sites of their recognition sequences varies, as also does the ‘reach’ to the cleavage site. However, if the methylated bases are taken as the reference points rather than the boundaries of the half-sites, then bi-lateral symmetry emerges reflecting the fact that the same RM subunit catalyzes the reactions on both sides.

Both subunits of BcgI-like enzymes (Type IIB) are required for cleavage activity and for methylation activity. The subunit stoichiometry is 2RM:1S (223), mirroring the 2M:1S stoichiometry of Type I MTases. The S subunits, though generally smaller than Type I S subunits, appear to have a similar organization of tandemly repeated specificity and dimerization domains. Unlike the monomeric Type IIG enzymes described in the previous section, BcgI-like enzymes protectively modify both strands of their recognition sequence without the need for accompanying MTases. The cleavage domain of the RM subunits contains only one catalytic site, and double-strand cleavage likely requires transient dimerization between neighboring enzyme molecules. Because cleavage takes place on both sides of the recognition sequence, multiple molecules are involved at the same time (228–231).

#### Cleavage geometry

As the RM subunits of Type IIB enzymes methylate the half-sequence to which they are bound, it seems logical that they would cleave on the same side, too. If so, the RM subunit would be cleaving on the 5′ side of the methylated base, upstream of the recognition site. Type IIG enzymes that bind continuous, rather than bipartite, recognition sequences invariably cleave on the 3′ side of the methylated base, downstream, in the opposite direction. The RM subunit of BcgI is similar in size and predicted structure to the corresponding RM domain of MmeI (described later in the text), suggesting that these two enzymes cleave and methylate with similar geometries. Nevertheless, BcgI cleaves 12/14 upstream, whereas MmeI cleaves 21/19 downstream (Table 4). How to reconcile this considerable catalytic discrepancy is not clear. One possibility is for the BcgI subunits to cleave on the other side of the recognition sequence, in which case they would be cleaving 21/19 downstream, exactly like MmeI. Another possibility is for MmeI to act as a dimer or tetramer and to cleave 12/14 upstream, but not of the sequence to which it is bound, but instead to a flanking, inverted sequence, that lies side-by-side.

#### Single-chain Type IIG enzymes: AloI

A number of single-chain Type IIG enzymes also cleave on both sides of bipartite recognition sequences. HaeIV, for example, cleaves on both sides of a recognition sequence that is symmetric, suggesting that it acts as a homodimer—the Type IIG equivalent of MTases like M.DrdI (Table 4) (232). In other enzymes, such as AloI and CjeI, the two subunits of a BcgI-like ancestor are fused into a single protein that possesses two TRDs at the C-terminus (233–235). These enzymes are ∼1250 aa in length, and both TRDs appear to be functional, as shown by domain-swap experiments (236). Yet, how they act is a mystery because the normal stoichiometry of 1RM:1TRD such as occurs in BcgI and all other Type II G enzymes, is reduced to 1RM:2TRD in CjeI and AloI (Figure 6). This reduction deprives one of the two TRDs of an RM domain with which to methylate or to cleave (226).

#### Bimodal Type IIG enzymes: MmeI

MmeI (TCCRAC 20/18) was the first Type IIG enzyme to be purified (237), but it was not well-characterized until many years later (238). MmeI belongs to a large family of closely related enzymes whose specificities have diversified extensively (196). ‘By-eye-oinformatics’ analysis correlating TRDs with sequences recognized, in combination with mutagenesis experiments, have led to the identification of most of the ‘contact’ amino acids responsible for sequence-recognition (196). Remarkably, when these aa are judiciously changed the specificities of the enzymes also change, predictably and robustly (196). A model of MmeI bound to DNA has been reported (239), and the X-ray crystal structures of MmeI bound to DNA, and of NmeAIII without DNA, have recently been solved [(195); Scott Callahan and Aneel Aggarwal, personal communication]. The DNA-bound TRD of MmeI superimposes on M.TaqI and the crystallized Type I S subunits (95,96), enabling the identities of some of the contact residues to be transferred. This might allow Type I R–M enzyme specificities to be changed rationally, base-by-base, in the same way they can now be changed in MmeI-family enzymes (196). Eventually, it might also allow the specificities of some of the thousands of the putative S subunits in REBASE to be assigned by simple inspection of their aa sequences.

MmeI-family enzymes purify as monomers, but it seems likely that *in vivo* they act as homodimers in somewhat the same way as Type III R–M enzymes are thought to do (240,241). MmeI-enzymes protectively modify their DNA without the need for accompanying MTases, and because their specificities are generally asymmetric, they methylate only one strand (238). This has led to the suggestion they be termed ‘Type IIL’ enzymes, for Lone-strand modification (242). It would be astonishing if the MmeI-family enzymes did rely on modification of only one strand for protection, however, because then they would have no way to distinguish unmodified, infecting DNA from unmodified, but newly replicated, host DNA. *A priori*, it seems likely that they rely instead on pairs of recognition sequences in opposite, head-to-head, orientations. One member of such pairs will always be modified in newly replicated DNA, whereas neither will be modified in infecting DNA.

Type I R–M enzymes faced this same problem during evolution and solved it by binding to two inverted half-sequences approximately one DNA helix turn apart. Things are somewhat different for the MmeI enzymes because the opposing sites are not a fixed distance apart, implying that protein–protein interaction must depend on sliding or DNA looping (243,244). MmeI-enzymes are predicted to have alpha-helical domains at their C-termini (239) that could act as dimerization zippers to interconnect adjacent enzyme molecules. Such helices occur at the C-termini of *γ*-MTases that act as dimers such as M.DrdI and M.AhdI, but they do not occur at the C-termini of *γ*-MTases such as M.TaqI that act as monomers and have no need for them (Figure 6). It seems plausible, then, that MmeI-type enzymes bind to their recognition sequences as monomers, but cleave DNA only after assembling into homodimers, the overall organizations of which are similar to Type I MTases. If so, what we refer to as the ‘recognition sequence’ of MmeI (i.e. TCCRAC) is the equivalent of a Type I half-sequence, and its complete binding site is the indefinite symmetric sequence: TCCRAC Ni GTYGGA (where i can be any number of base pairs between ∼50 and 5000).

#### ‘Type ISP’ enzymes

The recently christened ‘Type ISP’ enzymes combine features of both Type I and MmeI-type enzymes. They are long single-polypeptide chain fusions of, in order, an N-terminal Mrr-like endonuclease domain (typically, PD-QXK instead of PD-EXK), a DNA-translocase domain, a *γ*-MTase and a C-terminal TRD (245–247) (Figure 6). Type ISP enzymes recognize continuous, asymmetric sequences, or perhaps more accurately ‘half-sequences’, and they behave essentially like ‘half-Type I R-M enzymes’ showing ATP-dependent DNA translocation and a requirement for two target sites in head-to-head orientation for cleavage. Cleavage requires two molecules of the enzyme, and it occurs at a random location between the two sites (248). Translocation produces loops of DNA, but only a single recognition site, and a single copy of the enzyme, is needed for this to take place (249).

## CONCLUSION

Type I R–M enzymes and their relatives represent a huge group of sequence-specific DNA-binding proteins with varied specificities, oligomeric organizations and catalytic properties. Traditionally, they have been difficult to characterize, but genome sequencing, bioinformatics and, very recently, methylome analysis by next-generation SMRT sequencing have thrown open a wide door for identifying them and determining their target recognition sequences. As more of these enzymes are analyzed, a detailed understanding of how they recognize DNA will emerge. This could allow new specificities to be engineered at will, and new biochemical functions to be delivered to DNA sequences of choice. Type I enzymes and their relatives offer numerous opportunities for protein design and fabrication. Although they have been studied for >50 years, their potential as molecular tools and as sources of nano-scale biochemical components is only now becoming apparent.

## FUNDING

Funding for open access charge: New England Biolabs.

*Conflict of interest statement*. None declared.
